# Screening of Oligomeric (Meth)acrylate Vaccine Adjuvants Synthesized via Catalytic Chain Transfer Polymerization

**DOI:** 10.3390/polym15183831

**Published:** 2023-09-20

**Authors:** Cordula S. Hege, Amy Stimpson, Joseph Sefton, James Summers, Helena Henke, Adam A. Dundas, Tony Phan, Robert Kinsey, Jeffrey A. Guderian, Sandra J. Sivananthan, Raodoh Mohamath, William R. Lykins, Gabi Ramer-Denisoff, Susan Lin, Christopher B. Fox, Derek J. Irvine

**Affiliations:** 1 Centre for Additive Manufacturing, Department of Chemical and Environmental Engineering, University of Nottingham, Nottingham NG7 2RD, UKadam.dundas1@nottingham.ac.uk (A.A.D.); 2 School of Chemistry, University of Nottingham, Nottingham NG7 2RD, UK; 3 Access to Advanced Health Institute, Formerly Infectious Disease Research Institute, Seattle, WA 98102, USA; 4 Department of Global Health, University of Washington, Seattle, WA 98104, USA

**Keywords:** polymerization, screening, catalytic chain transfer, vaccine, adjuvant, squalene

## Abstract

This report details the first systematic screening of free-radical-produced methacrylate oligomer reaction mixtures as alternative vaccine adjuvant components to replace the current benchmark compound squalene, which is unsustainably sourced from shark livers. Homo-/co-oligomer mixtures of methyl, butyl, lauryl, and stearyl methacrylate were successfully synthesized using catalytic chain transfer control, where the use of microwave heating was shown to promote propagation over chain transfer. Controlling the mixture material properties allowed the correct viscosity to be achieved, enabling the mixtures to be effectively used in vaccine formulations. Emulsions of selected oligomers stimulated comparable cytokine levels to squalene emulsion when incubated with human whole blood and elicited an antigen-specific cellular immune response when administered with an inactivated influenza vaccine, indicating the potential utility of the compounds as vaccine adjuvant components. Furthermore, the oligomers’ molecular sizes were demonstrated to be large enough to enable greater emulsion stability than squalene, especially at high temperatures, but are predicted to be small enough to allow for rapid clearance from the body.

## 1. Introduction

Squalene (and/or squalane, the fully hydrogenated derivative), which is a key adjuvant component in several commercially licensed vaccines such as influenza vaccines [1,2,3], enhances vaccine potency, thus allowing dose sparing of the vaccine antigen [4]. Squalene is a naturally occurring material; however, it is mainly derived from shark livers, limiting its sustainability. It can also be extracted from agricultural sources [5,6], yet these sources prove to be expensive and process-intensive as the quantities are too small [7]. Thus, the development of synthetic routes is urgently needed to produce alternative adjuvant components and to reduce this reliance on natural squalene.

We have previously shown that squalene analogs can be produced using free-radical polymerization and isoprene as the monomer and that these analogs are capable of eliciting vaccine adjuvant effects on human cells [8,9]. In this study, which compared the application of different polymerization control systems to produce oligomers, it was concluded that catalytic chain transfer polymerization (CCTP) was the most appropriate method of control to apply based on yield and the quantity of the control agent required [8,9]. Furthermore, (meth)acrylate compounds, which can be polymerized using free-radical chemistry, have been shown to have some utility as vaccine adjuvants. For example, poly(acrylic acids) and poly(methacrylates) have been used as vaccine adjuvants [10,11,12]. Additionally, polymers produced via the γ-radiation of methyl methacrylate, methyl acrylate, and butyl methacrylate have been tested for vaccine adjuvant activity, with poly(methyl methacrylate) (PMMA) variants exhibiting small particle sizes found to be the most immunogenic [13]. Poly(methacrylate)-derived adjuvants have been employed as adjuvants for inactivated HIV antigens and have strengthened the antibody response and protective efficacy of influenza vaccines [11,13]. More recently, polyacrylate esters have demonstrated utility as components of peptide conjugate vaccine candidates [14,15,16]. Moreover, non-crosslinked polyacrylate polymers have demonstrated similar or superior adjuvant activity compared with squalene emulsions in mice and non-human primates immunized with recombinant vaccine antigens, as well as in human cells using an in vitro innate immune module, with the adjuvant activity being dependent on polymer molecular weight [17,18].

Control over the molecular weight of final products is important to ensure that the materials can be formulated into emulsions (and that they exhibit sufficiently low viscosity) and to retain other key biological properties. For example, polymers that are required to undergo renal clearance should be of a low molecular weight because the speed of linear polymer renal clearance decreases rapidly from 19 to 70 kg/mol [19,20]. In recent decades, new methods for controlling free-radical polymerization have been developed, including reversible addition–fragmentation chain-transfer (RAFT) polymerization, atom transfer radical polymerization (ATRP), and nitroxide-mediated polymerization (NMP) [21,22,23]. However, with these techniques, it can be difficult to synthesize very low-molecular-weight polymers, often referred to as oligomers. CCTP is a technique that is well-known for the synthesis of methacrylate oligomers [24]. Additionally, unlike RAFT, ATRP, and NMP, the CCTP mechanism introduces a vinyl functionality into the product as a terminal group, which can act as a site for post-reaction or post-functionalization [25,26,27].

In practice, suitable CCTP catalysts are metal-based complexes capable of a kinetically labile ± 1 oxidation state change [28]. Cobalt cobaloxime complexes are active catalysts for CCTP, and those that contain BF_2_ moieties that bridge between the glyoxime ligands or those derived in a single stage from halide complexes have enhanced stability and improved efficacy [24,29,30]. Consequently, they are the most commonly used catalysts for CCTP, with the most predominant variants containing methyl or phenyl groups in the ligand systems [27,31,32,33]. However, when synthesizing materials for commercial applications, the use of cobalt as a metal center is a potential disadvantage of CCTP, even if it is present at only ppm or ppb levels. This is because cobalt is a rare element with a frequency in the Earth’s crust of 0.004%, which is approximately 2500 times less than iron. Consequently, there have been reports in the literature of the use of a range of alternative iron-based complexes that act as either ATRP or CCTP catalysts depending on the ligand used [34,35,36].

The aim of this study was to demonstrate the successful screening of the material and biological performance of a range of (meth)acrylate mixtures containing oligomers of controlled molecular weights, and thus to inform the subsequent design and synthesis of adjuvant alternatives to squalene. Consequently, in this first study, commercially available methacrylate monomers were used to prepare oligomer mixtures that were screened for use as vaccine adjuvant components. The ability of these mixtures to generate emulsions demonstrating appropriate particle sizes, physicochemical stability, and in vitro innate immunomodulatory activity in human primary cells was evaluated. More specifically, the use of both cobalt- and iron-centered catalysts was examined to determine if they can be used to produce low-molecular-weight (meth)acrylates following CCTP, either as isolated catalysts or when generated in situ. Furthermore, the influence of microwave heating was investigated.

## 2. Materials and Methods

### 2.1. Materials

Methyl methacrylate (MMA, 99%), ethyl methacrylate (EMA, 99%), butyl methacrylate (BMA, 99%), lauryl methacrylate (LMA, 96%), stearyl methacrylate (SMA, 89.5%), methyl acrylate (MA, 99%), iron(II) bromide (FeBr_2_, 98%), 2,2-azobis(isobutyronitrile) (AIBN, 98%), glyoxal solution (40 wt% in H_2_O), 2,6-diisopropylaniline (97%), 2,4,6-trimethylaniline (98%), and shark squalene were purchased from Sigma-Aldrich (Gillingham, UK). Cobalt(II) bromide (CoBr_2_, 98%) and dimethyl glyoxime (DMG, 99%) were purchased from Acros Organics (Verona, Italy). Diphenyl glyoxime (DPG, 99%) was purchased from Alfa Aesar (Heysham, UK). Bis[(difluoroboryl) diphenylglyoximato]cobalt(II) (PhCoBF) was supplied by DuPont (Wilmington, DE, USA). Technical-grade toluene, propan-2-ol, and diethyl ether were obtained from campus departmental stores. Amaranth squalene was purchased from Wilshire Technologies (Princeton, NJ, USA). Napa Valley Naturals (Stonewall Kitchen, York, ME, USA) grapeseed oil was purchased from a local grocery store. 1,2-dimyristoyl-sn-glycero-3-phosphocholine (DMPC) was obtained from Lipoid (Ludwigshafen, Germany). Poloxamer 188 and glycerol were obtained from Spectrum Chemical (New Brunswick, NJ, USA). Buffer components were obtained from J.T.Baker (Phillipsburg, NJ, USA) or Fluka (Charlotte, NC, USA). All materials were used as supplied without further purification.

### 2.2. Synthetic Procedures

Diimine Ligand Synthesis

Glyoxal solution (10 g, 0.17 mol), propan-2-ol (16 mL), and water (40 mL) were added to a solution of 2,6-diisopropylaniline (61.09 g, 0.34 mol) or 2,4,6-trimethylaniline (46.59 g, 0.34 mol) dissolved in propan-2-ol (160 mL) under an argon atmosphere, and the reaction vessel was submerged in a preheated oil bath at 70 °C for 1 h. After the reaction was complete, the reaction vessel was cooled, deionized water (160 mL) was added to the reaction medium, and a yellow precipitate formed, which was collected via filtration and dried under vacuum.

### 2.3. Typical Polymerization Procedures to Produce Oligomer Mixtures for Screening

#### 2.3.1. Pre-Isolated Cobalt Catalyst Polymerization Method

MMA (10 mL, 93.49 mmol) and toluene (10 mL) were degassed and transferred to a reaction vessel containing the appropriate amounts of PhCoBF and AIBN (94 mg, 0.57 mmol) under an inert atmosphere. In conventionally heated reactions, the reaction vessel was submerged into a preheated, thermostatically controlled oil bath pre-set to 80 °C for 3 h, after which the reaction was quenched by placing the vessel in an ice bath. The polymers for material analysis were purified by exhaustively washing with water and then filtering using a Fisherbrand filter paper (Grade 601) into vials for storage. Meanwhile, the samples for performance assays, which were the products of solventless bulk reactions, were filtered directly into Falcon tubes and dispatched as mixtures.

#### 2.3.2. In Situ Catalyst Method for Both Cobalt- and Iron-Based Polymerization

MMA (10 mL, 93.49 mmol) and toluene (10 mL) were degassed and transferred to a reaction vessel containing the appropriate amounts of FeBr_2_ and the chosen ligand, i.e., [RN=CH-CH=NR] (R = 2,6-diisopropylphenyl (DIIP) or 2,4,6-trimethylphenyl (TMP)), DMG or DPG, and AIBN (94 mg, 0.57 mmol) under an inert atmosphere. The remainder of the procedure is detailed in the pre-isolated cobalt catalyst polymerization method above.

For the biological assays, both homo- and co-polymers were produced in the desired molecular weight range by utilizing either 5000, 7000, or 10,000 ppm of AIBN and catalyst-ligand loadings of 1250, 2500, or 6000 ppm. The chosen monomers were MMA, BMA, LMA, and SMA.

#### 2.3.3. Example for Homopolymer Synthesis: Polymerization of LMA

LMA (23 mL, 78.6 mmol) was degassed and transferred to a reaction vessel containing CoBr_2_ and the chosen ligand, either DMG or DPG, and the appropriate amount of AIBN under an inert atmosphere. The remainder of the procedure is detailed in the pre-isolated cobalt catalyst polymerization method above.

#### 2.3.4. Example for Copolymerization: Polymerization of Poly(lauryl methacrylate-co-butyl acrylate)

LMA (17.3 mL, 58.96 mmol) and BMA (16.77 mL, 105 mmol) were degassed and transferred into a reaction vessel containing CoBr_2_ and the chosen ligand, either DMG or DPG, and the appropriate amount of AIBN under an inert atmosphere. The remainder of the procedure is detailed in the pre-isolated cobalt catalyst polymerization method above.

#### 2.3.5. Microwave- Versus Conventionally Heated Polymerizations

The conventionally heated reactions were conducted as described in the pre-isolated cobalt catalyst polymerization method above. In the microwave-heated reaction procedure, the reaction vessel was placed into the cavity of a Sairem (Décines-Charpieu, France) MiniFlow 200SS with the target temperature set to 80 °C, which was monitored using an optical fiber probe inserted within a glass sheath through a septum into the bulk of the liquid. The power was set to 200 W at a frequency of 2.45 GHz for 3 h. The polymers were isolated and purified as described in the pre-isolated cobalt catalyst polymerization method above.

### 2.4. Characterization Methods

#### 2.4.1. Gel Permeation Chromatography (GPC)

GPC analysis was performed using an Agilent 1260 Infinity instrument (Agilent Technologies, Santa Clara, CA, USA) equipped with a double detector with the light scattering configuration. Two mixed C columns at 35 °C were employed, using tetrahydrofuran (THF) as the mobile phase with a flow rate of 1 mL/min. GPC samples were prepared in HPLC-grade THF and filtered before injection. The analysis was carried out using ASTRA version 6.1 (Wyatt Technology Corporation, Santa Barbara, CA, USA) software. The number and weight average molecular weight (M_n_ and M_w_, respectively) and polydispersity (Ð) were calculated using narrow standards of PMMA for the calibration curve. The GPC standards used were purchased from Agilent as the InfinityLab EasiVial PMMA pre-weighed calibration kit with samples covering the range from M_n_ ~600 to ~2,000,000.

#### 2.4.2. Proton Nuclear Magnetic Resonance (^1^H NMR) Spectroscopy

NMR spectra were recorded at 25 °C using Bruker (Billerica, MA, USA) AV400 and AV3400 spectrometers (400 MHz) and deuterated solvents. Chemical shifts were assigned in ppm. Samples were prepared as solutions in deuterated chloroform (CDCl_3_), to which chemical shifts were referenced (the δH of residual chloroform at 7.26 ppm). Mnova version 14.2.1 (Mestrelab Research, S.L., Santiago de Compostela, Spain) was used to analyze the spectra. Polymerization conversions were calculated via integration and the comparison of the methylene group from the ester group of the monomer and polymer.

#### 2.4.3. Emulsion Formulation and Physical Stability Assessment

The synthesized oligomer solutions were formulated with two emulsifiers (0.76% *w*/*v* DMPC and 0.036% *w*/*v* poloxamer 188), 2.3% *w*/*v* glycerol, and a 25 mM ammonium phosphate buffer (pH 5.8) and were processed via high-shear mixing and high-pressure homogenization to produce oil-in-water nanoemulsions containing 4% *v*/*v* oil (squalene, triglyceride, or poly(acrylate) material) as described previously [37,38]. In addition to the methacrylate-based polymers, emulsions were prepared using shark-derived squalene, amaranth-derived squalene (i.e., squalene derived from plant sources), and a long-chain triglyceride for comparison [37]. For a commercial product benchmark comparator in the immunogenicity study, a squalene emulsion representing the MF59 composition was prepared, containing 4% *v*/*v* shark squalene, 0.41% *w*/*v* polysorbate 80, 0.41% *w*/*v* sorbitan trioleate, and 10 mM citrate (pH 6.0), via high-shear mixing and high-pressure homogenization to generate a droplet diameter of 150 nm. The emulsion droplet diameter and polydispersity index (PDI) were monitored using dynamic light scattering using a Zetasizer-S, -ZS, or -APS (Malvern Panalytical, Malvern, UK) as previously described [38], with storage at 5 °C, 25 °C, and 40 °C for up to 18 months.

#### 2.4.4. Peripheral Blood Mononuclear Cell (PBMC) Viability Assay

PBMCs isolated from a human male donor were resuspended at 0.5 × 10^6^ cells/mL in Roswell Park Memorial Institute (RPMI) medium with 10% fetal calf serum (FCS). The collection of human blood products via standard venipuncture or leukapheresis was performed under the ICH Guidelines for Good Clinical Practice and US CFR Title 45 Part 46 (Protection of Human Subjects). The research protocol (WIRB Protocol 20020527) and the associated informed consent document were reviewed and approved by the Western Institutional Review Board. All research subjects provided written informed consent prior to the study procedures.

The selected emulsions (0.1–0.4% oil diluted in RPMI and 10% FCS) were mixed with 1.0 × 10^5^ PBMCs in 96-well U-bottom plates and incubated at 37 °C for 18 h. The mixtures were then diluted 1:20 *v*:*v* with Guava ViaCount Reagent (Luminex, Austin, TX, USA) and incubated in the dark for 5 min at ambient temperature. Viability was measured using the Guava easyCyte HT (Luminex).

#### 2.4.5. In Vitro Innate Immune Stimulation Activity

Stable emulsions (SEs) were evaluated for innate immunostimulatory activity in whole blood from 6 human subjects (3 male and 3 female) that was purchased anonymized from Bloodworks Northwest (Seattle, USA). The emulsions were incubated with heparinized blood for 18–24 h at 37 °C, and the production of monocyte chemoattractant protein-1 (MCP-1), interleukin 8 (IL-8), and macrophage inflammatory protein-1β (MIP-1β) cytokines was quantified in the supernatants as described previously [9,39]. These cytokines were selected based on their robust modulation with squalene emulsions in our previous studies.

#### 2.4.6. In Vivo Study

C57BL/6 (B6) mice were purchased from The Jackson Laboratory (Harbor, USA). The experimental groups consisted of 6–8-week-old male and female mice (*n* = 7–8). The mice were immunized via intramuscular injection of a 100 µL total volume (50 µL in each hind leg) of the vaccine containing 10 ng of split, inactivated H5N1 A/Vietnam/1194/2004 (National Institute for Biological Standards and Control, Potters Bar, UK), and a 2% *v*/*v* oil-in-water emulsion on Study Day 0 and Study Day 21. All animal experiments were performed in accordance with national and institutional guidelines for the animal care of laboratory animals and approved by the Infectious Disease Research Institute Institutional Animal Care and Use Committee. Results from the control groups were previously reported as part of a concurrent study [40].

Peripheral blood was collected on Study Day 42 via cardiac puncture after euthanasia. Blood was collected in Sarstedt (Nümbrecht, Germany) Microvette capillary blood collection tubes and centrifuged to separate the serum. The serum was stored at −20 °C until analysis. On Study Day 42, the mice were euthanized via the controlled administration of carbon dioxide inhalation, followed by cervical dislocation, and spleens were removed aseptically. Lymphocytes were isolated from the spleens using manual disruption. Red blood cells contained in the spleens were lysed using Red Blood Cell Lysis Buffer (eBioscience, San Diego, CA, USA). The lymphocytes were used for cytokine (interferon-γ (IFNγ) and interleukin 5 (IL-5)) enzyme-linked immunospot (ELISpot) assays as described below.

Antigen-specific total immunoglobulin G (IgGT), IgG1, and IgG2c were measured in the serum samples from the immunized animals using enzyme-linked immunosorbent assay (ELISA) and antibodies purchased from Southern Biotech (Birmingham, USA, #1031-05) that were diluted 1:5000, 1:3000, and 1:2000, respectively. Briefly, 384-well plates were coated with 1 µg/mL of recombinant H5 A/Vietnam/1194/2004 antigen (Sino Biological, Chesterbrook, PA, USA) overnight. The next day, the plates were washed and blocked with phosphate-buffered saline (PBS) with 0.1% polysorbate 20 and 1% bovine serum albumin (BSA). After washing, plates were incubated with the serum followed by incubation with horseradish peroxidase (HRP)-conjugated antibodies and 3,3′,5,5′-tetramethylbenzidine (TMB) substrate. The reaction was stopped using 1 N H_2_SO_4_ and read within 30 min using a Victor X4 (PerkinElmer, Waltham, MA, USA) plate reader. Endpoint titers were interpolated using a sigmoidal curve fit and an arbitrary cutoff value of 0.5 or 0.75 depending on the background signal. Endpoint titers below the standard curve range were assigned the value corresponding to 0.5× of the lowest standard curve dilution.

ELISpot plates were coated with IFNγ (BD Biosciences, Franklin Lakes, NJ, USA, #551881 or eBioscience #88-7384) and IL-5 (BD Biosciences #551880 or eBioscience #88-7825) capture antibodies, diluted 1:200, and stored overnight at 4 °C. The plates were washed with PBS–Tween (PBST), blocked with complete RPMI medium for 2 hours at ambient temperature, and then washed again. Splenocytes were plated at 2.0 × 10^5^ cells per well and were stimulated with 10 µg/mL of recombinant H5 A/Vietnam/1194/2004 antigen (Sino Biological) at 37 °C with 5% CO_2_ for 48 hours. The plates were washed with PBST, detection antibodies (BD Biosciences #551881 and #551880 or eBioscience #88-7384 and #88-7825), diluted 1:250, were added, and the plates were then incubated overnight at 4 °C. After incubation, the plates were washed with PBST, and Avidin D (Av)-HRP (Invitrogen, Carlsbad, CA, USA, #50-112-3249), diluted 1:250, was added for a 45 min incubation at ambient temperature followed by a PBS wash. The plates were developed with 3-amino-9-ethylcarbazole (AEC) substrate kits according to the manufacturer’s protocol. The reaction was stopped by washing the plates with deionized water, the plates were dried in the dark, and spots were counted using an automated ELISpot reader (CTL Analyzer, Cellular Technology Limited, Cleveland, OH, USA). Data were analyzed using ImmunoSpot version 7 professional software (Cellular Technology Limited).

Log-transformed IgGT antibodies, cytokine-secreting cell counts, and ratios were analyzed using one-way ANOVA with Tukey’s correction for multiple comparisons. Log-transformed IgG1 and IgG2c included non-normally distributed data and were thus evaluated using the non-parametric Kruskal–Wallis test with Dunn’s correction for multiple comparisons.

## 3. Results and Discussion

Principally methacrylate monomers were chosen for this study to build CCTP-generated oligomers. To determine if these CCTP-generated oligomer mixtures were potential adjuvant candidates, their biocompatibility, emulsion stability, and bioactivity were evaluated. To try to maximize their potential biocompatibility, we investigated the use of either (a) ppm levels of cobalt catalysts as control catalysts for the polymerization or (b) iron catalysts as CCTP catalysts. Both methods were then compared with standard, initiator-controlled free-radical “blank” polymerization (i.e., containing no CCTP catalyst). The efficiency of the catalysts was determined by comparing the molecular weight, polydispersity (Ð), and percent conversion of the oligomer obtained from the metal-mediated reactions to that of the materials produced from the “blank” polymerization.

We previously showed the successful generation and use of in situ cobalt catalysts to achieve good CCTP control [32,33]. These catalysts were generated within the polymerization media prior to polymerization by adding CoBr_2_ and DPG or DMG ligands individually. This in situ cobalt methodology successfully achieved the generation of oligomers without the need to pre-stir the catalyst reagents upon addition to the polymerization medium [32,33]. This was demonstrated to be an advantageous method as it removed issues with catalyst synthesis, storage, and costs. Thus, we adopted a similar in situ strategy in this study to benchmark the performance of the iron catalysts.

We previously generated poly(isoprene) oligomers via CCTP that were shown to generate stable oil-in-water emulsions with adjuvant activity [9]. In this prior work, two series of oligomers were synthesized as squalene analogs and evaluated for emulsion stability. Compared with squalene (M_n_ = 411 g mol^−1^; degree of polymerization (DP) = 6) and squalane (M_n_ = 423 g mol^−1^; DP = 6), these analogs were in the molecular weight ranges of (a) 530–660 g mol^−1^ (~DP = 8–10) and (b) 2100–3000 g mol^−1^ (~DP = 30–44). The former produced nanoemulsions; however, they were susceptible to emulsion droplet diameter growth over time, possibly due to Ostwald ripening. Meanwhile, emulsions formulated from the latter demonstrated stability profiles at 2–8 °C that were more comparable to those of shark squalene. Thus, the initial target for the CCTP oligomer production was to make polymers in the ~3000 g mol^−1^ region to hopefully ensure that Oswald ripening was not observed from the samples created in this study.

In the present study, our initial goal was to synthesize methacrylates that did not have unsaturation in their pendant groups to avoid unsaturation playing an active role in the CCTP. Therefore, the monomers chosen were MMA, EMA, BMA, LMA, and SMA (Figure S1). These gave a significant range of pendant group chain lengths and thus glass transition temperature (T_g_) values for the resulting oligomers.

### 3.1. Results with Cobalt-Catalyzed CCTP

Reactions were initially optimized using the cobalt-catalyzed CCTP method. Table 1 (see also Figure S2) details the oligomers that resulted from these reactions, where the estimated degree of polymerization (EDp) is provided to enable a better comparison between the oligomers with varying repeat unit sizes. All cobalt catalysts were very effective control agents for the polymerization of the methacrylate monomer chosen and compared well to previously reported data [32]. However, due to the presence of the catalyst, the conversion of reactions was reduced due to the inhibition of the polymerization by the control agent. Thus, the percent conversion was determined for all oligomer mixtures produced, as it defined the concentration of the polymer within the mixtures.

### 3.2. Results with Iron-Catalyzed CCTP

We conducted an initial benchmarking study to determine if iron catalysts could control the oligomerization and, if so, whether the levels of the control agent required to deliver oligomers of similar molecular weight were similar to those in cobalt-catalyzed CCTP (Table 1). Again, we used MMA with 600 and 6000 ppm catalyst loadings of either the isolated catalyst or in situ catalyst, where the pure catalyst reagents were not pre-stirred prior to polymerization [32,33]. In this study, the complexes were chosen to follow the design from prior work [34], namely, a metal-pre-catalyst-to-ligand molar ratio of 1:1. Figure S3 shows the proposed molecular structures of the resulting catalyst–ligand complexes formed with the DIPP, TMP, DMG, or DPG ligands used. The isolated iron catalysts were synthesized from the appropriate iron(II) halide precursor and one of two different ligands, either DIPP (L1) or TMP (L2). Meanwhile, DMG (L3) and DPG (L4) ligands were only used in the in situ method to mimic prior cobalt studies [32,33]. Again, toluene was used as the solvent for the oligomerizations.

Table 2 details the results with either 600 or 6000 ppm loadings of the catalysts and ligands. The results show that the iron catalysts exerted control over the polymerization compared with the “blank” reaction, but they were significantly less efficient than the cobalt chain transfer agents (CTAs). Furthermore, isolating the catalysts before the reaction did not increase control of the reaction with either loading, which demonstrated that the in situ strategy was a valid method for producing these specific iron-based CTA complexes within a polymerization medium. All the experiments produced polymers that were in the ~20,000 to 37,000 g mol^−1^ range compared with the 300 to 500 g mol^−1^ range that was achieved with the in situ cobalt alternatives (Table 1). Furthermore, using 6000 ppm of iron catalysts only led to a relatively small increase in the control of the reaction compared with 600 ppm. This suggested that it could take longer to generate the Fe catalyst as there might not be enough iron present.

### 3.3. Comparison of Conventionally Heated with Microwave-Heated Experiments

The application of microwave heating (MWH) to the FeBr_2_-controlled MMA polymerizations was investigated to determine if this would increase the efficiency of catalyst generation and thus the level of control over the reactions. This mode of energy input can significantly reduce the time required for these organometallic reactions to occur due to the selective heating of the metallic reagents [41]. However, with pre-synthesized cobalt catalysts, the use of MWH in cobalt-based CCTP has also led to propagation being promoted over chain transfer, resulting in an increased M_n_ [30]. This was attributed to the microwaves selectively heating the radical dipole preferentially to the cobalt precursors/complexes. Alternatively, with in situ catalysts, reductions in M_n_ values were observed as it was proposed that the more rapid temperature rise in the medium resulted in the more efficient formation of the “true” catalyst [33]. Additionally, whilst cobalt is paramagnetic, iron is ferromagnetic. Therefore, we wanted to determine if this promotion of propagation was also observed in the iron-based CCTP. Thus, the three potential outcomes that may arise from applying MWH in this study are (1) an increase in the efficiency of the catalyst formation and chain transfer from selectively heating the iron species, which increases the reaction inhibition and reduces the M_n_, (2) an increase in the rate of propagation by selectively heating the propagating polymer radicals, leading to an increase in the M_n_ as observed in the cobalt case, or (3) both species selectively heating, leading to a reduction in both the M_n_ and polymerization time. For ease of preparation, we chose the in situ polymerizations for the MWH study and a 1:1 metal-to-ligand ratio to minimize the amount of catalyst synthesis required to achieve the formation of the active complex.

The results of the MWH study are shown in Table 3. In all cases, while some control was clearly exerted over the system, as the MWH polymers exhibited lower M_n_ and Ð values than those obtained from the uncontrolled “blank” reaction (Table 1, Entry 1), applying MWH did not enhance the reaction control. Instead, it led to an approximately two-fold increase in molecular weight in comparison with conventional heating, suggesting that an increase in the propagation rate occurred due to the selective heating of the polymer radical moiety, as has been observed previously [32]. The reduction in control was also evidenced in the conversion achieved. All MWH conversions were above 80%, indicating little catalyst-based inhibition of the MWH polymerization.

### 3.4. Optimization of Iron-Catalyzed CCTP Reactions

Consequently, an extensive series of conventionally heated in situ iron experiments was then conducted with the iron-catalyzed systems to optimize the reaction conditions for these systems. Commercially available DMG and DPG ligands were used because they were available in large quantities. In these experiments, the monomer type was varied. The key optimized reactions from this series of experiments are shown in Table 4.

In general, these experiments demonstrated that the use of DMG as a ligand did not exert any significant control over the polymerization. For comparison, see the very similar results recorded for the MMA oligomerization with FeBr_2_ and DMG (Table 4, Entry 1) and the catalyst-free uncontrolled reaction conducted using these screening methodologies (Table 1, Entry 1). Meanwhile, using DPG as the ligand demonstrated (1) molecular weight reductions to produce a polymer with similar Ð values (compare Table 4, Entry 1 and Entry 4) and (2) that the side-chain length exerted some influence on the ED_p_ achieved. The ED_p_ decreased with an increasing side-chain length (Table 4, Entries 4 to 6), suggesting that the catalysts were more efficient with monomers containing larger pendant groups. This was attributed to the slower polymerization rates exhibited by these more sterically cluttered monomers, thus potentially allowing more time for the catalyst to be synthesized and for it to assert control via chain transfer.

### 3.5. CCTP of Acrylates

We conducted an additional evaluation with the iron catalysts to determine if these catalysts had any utility with acrylates. As the methacrylate adjuvant mixture systems that proved to show a positive emulsion assay performance (see below) contained polymers known to exhibit low T_g_ values (i.e., BMA = 20 °C and LMA = −65 °C) [42], the ability to make acrylate-based oligomers may be of potential interest. CCTP typically does not exhibit high levels of efficiency with these monomers [28]. Thus, the benchmark catalyst (PhCoBF) and both the isolated and in situ DIPP catalyst were applied to the polymerization of methyl acrylate (MA). For this series, the DIPP complex was chosen due to the high reactivity of this monomer.

The results of these reactions are detailed in Table 5. When no control agent or PhCoBF was used in the polymerizations of MA, even at high concentrations (i.e., 600 or 6000 ppm), a Trommsdorff–Norrish runaway was observed within 20 min of the reaction commencing. Meanwhile, when the iron catalyst systems were applied, a definable level of control was exhibited with this monomer as the reactions did not produce a Trommsdorff–Norrish effect. In the case of the pre-isolated complexes, a similar amount of CTA was required to control the MMA monomer (6000 ppm; see Table 2, Entry 9) and the acrylate equivalent. Both the in situ and isolated 600 ppm catalyst loadings showed similar levels of control to one another but had higher Ð values than the 6000 ppm experiment, and these higher Ð values also resulted in an unexpectedly lower M_n_. Overall, the iron system seemed to have greater utility for controlling monomers that do not contain an alpha-methyl group next to the polymerizable double bond compared with the equivalent cobalt complexes. However, at this point, the level of control was insufficient to produce acrylate oligomers of the target molecular weight required for adjuvant end-use and therefore will be the subject of further study.

### 3.6. Scale-Up of the Methacrylate Polymer Mixtures

Larger quantities of the oligomer mixtures were then generated to evaluate their utility as vaccine adjuvant components. To achieve larger volumes, these reactions were conducted in bulk (solventless) with the goal of synthesizing oligomers that had an ED_p_ and M_n_ similar to squalene and previously generated poly(isoprene) oligomers [9]. The oligomer mixtures with these features are shown in Table 6 (see also Figures S4–S9) and were subsequently tested for their potential use as adjuvant components.

These candidate mixtures were evaluated for both their ability to create and stabilize oil-in-water emulsions and their in vitro innate immune stimulation activity. In these evaluations, the synthesized oligomer mixtures were used as supplied with no further purification to create the test formulations. In the emulsion assays, all mixtures in Table 6 were formulated with DMPC, poloxamer 188, glycerol, and a 25 mM ammonium phosphate buffer (pH 5.8). Meanwhile, for the in vitro bioassays, all mixtures in Table 6 were utilized except for the MMA (Table 6, Entry 4) and SMA/LMA co-oligomer (Table 6, Entry 10) mixtures, which did not formulate due to their higher viscosity.

### 3.7. Emulsion Formulation and Physical Stability

The synthesized oligomer mixtures were formulated with DMPC, poloxamer 188, glycerol, and a 25 mM ammonium phosphate buffer (pH 5.8) as described above. The properties of these emulsions were then compared with emulsions prepared using shark-derived squalene, amaranth-derived squalene, and a long-chain triglyceride. In this case, the shark squalene emulsion was the positive control, whereas the long-chain triglyceride emulsion was the negative control in the in vitro biological activity assays below because it exhibits an absence of immunostimulatory properties [39].

**Figure 1:**
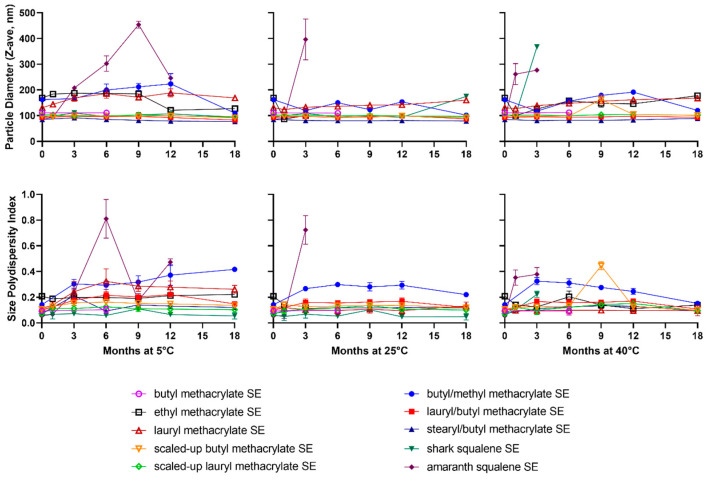
Physicochemical stability of oil-in-water emulsions resulting from methacrylate oligomer mixtures (Table 6). Stable emulsions (SEs) were stored at 5 °C, 25 °C, or 40 °C for up to 18 months and monitored using dynamic light scattering to assess emulsion droplet diameter (**top**) and size polydispersity index (**bottom**). Data are represented as mean values with error bars that represent the standard deviation. The MMA (Table 6, Entry 4) and the SMA/LMA co-oligomer (Table 6, Entry 10) mixtures did not formulate due to the higher viscosity of these oligomer mixtures and were therefore excluded.

The emulsion droplet diameter and polydispersity were monitored after storage at 5 °C, 25 °C, and 40 °C for 18 months. In general, representative size distributions measured immediately after emulsion manufacture indicated unimodal distributions (Figure S10). Several interesting stability trends were observed within these data, the most obvious being that the emulsion made with amaranth-derived squalene (agriculturally sourced) was physically unstable at all temperatures, whereas the shark-derived squalene emulsion demonstrated little or no change in particle size or polydispersity index (PDI) for at least 18 months at 5 °C and 12 months at 25 °C (Figure 1). Although not evaluated in the current study, it is possible that this discrepancy in stability could be attributable to different impurity contents in the oils obtained from different sources; for example, a previous report indicated a different impurity profile for olive-derived squalene compared with shark squalene [5]. These findings highlight the importance of raw material sources and the difficulty of finding suitable replacements for shark squalene.

Successfully formulated methacrylate-based emulsions demonstrated droplet sizes below the key threshold value of 200 nm. Below this value, particles can pass through a terminal 0.22-µm filter used for sterilization. In fact, a number of the methacrylate-based emulsions exhibited initial droplet sizes similar to that of the shark squalene emulsion (Figure 1 top). Meanwhile, the shark squalene emulsion generally demonstrated the smallest initial PDI, although many of the methacrylate-based emulsions’ PDIs were also below 0.2 (Figure 1 bottom). This value indicates that the samples exhibited mostly monodisperse populations. Furthermore, even the emulsions with larger initial droplet sizes appeared to maintain physical stability over time, although some emulsions decreased in droplet size, which was unexpected (Figure 1 top). In fact, several methacrylate-based emulsions demonstrated remarkable physical stability even at 40 °C, which was the most stringent condition applied. Moreover, the methacrylate-based emulsions typically appeared to be more physically stable than the shark squalene emulsion at 40 °C, which has potentially beneficial implications for the long-term storage of these emulsions. A closer investigation of these data suggested that the following methacrylate materials exhibited the best combinations of droplet properties: BMA, 22 mL scaled BMA, 21 mL scaled LMA, LMA/BMA, and SMA/BMA.

Detailed observation of the formulation data suggested that the apparent viscosity of the oligomer mixtures significantly influenced the emulsion results. In general, the apparent viscosity of the overall oligomer mixture was observed to show a strong dependency on the material properties of the whole mixture, in particular, the T_g_ of the polymeric material of construction, the molecular weight of the oligomer, and the degree of conversion achieved. In practice, these properties are interlinked; in principle, the repeat unit molecular structure will define the T_g_ of the polymer of construction (e.g., MMA = 105 °C, EMA = 65 °C, BMA = 20 °C, LMA = −65 °C, and SMA = −100 °C) [42]. However, this T_g_ value will be affected by the molecular weight achieved, with oligomers that are below the entanglement length of the polymer of construction exhibiting a reduction from the “theoretical” value for that polymer [48]. This is because the smaller chains can achieve a greater degree of relative intermolecular motion. Also, with oligomers, which possess significantly more chain ends than higher-molecular-weight polymers, the free volume associated with the chain ends will also influence the T_g_ observed [48]. Additionally, in the specific case of SMA, oligomers may also exhibit semi-crystalline characteristics, which may also influence the thermal behavior exhibited by these materials. Finally, the conversion achieved (Table 6) will also affect the oligomer mixture’s apparent viscosity, as the very low-molecular-weight monomers present will act as “plasticizers”/solvents again lowering the apparent oligomer T_g_ from the “theoretically” reported value. Unfortunately, the experimental measurements of the viscosity of these methacrylate oligomer mixtures and the “true” T_g_ values of the oligomers proved to be highly problematic/unreliable. The former was due to monomer evaporation during the timeline of the measurement, and the latter because of their physical form (i.e., sticky solids) and the high boiling point of the monomers, making the normal method of purifying small-scale samples (i.e., precipitation and vacuum drying) ineffective with these materials. Thus, these measurements were not conducted during this screening study. The next stage of this work will investigate routes to scale up and purify these target materials to determine these properties.

In terms of the particle size data obtained, typically, emulsions with oligomer mixtures formulated from monomers that “theoretically” gave rise to polymers with higher T_g_ values were either not processable (i.e., the methyl derivative (MMA; Table 6, Entry 4)) or produced larger particles across all temperatures tested (i.e., the butyl/methyl co-oligomer (BMA/MMA; Table 6, Entry 7) and ethyl oligomer (EMA; Table 6, Entry 1) mixtures). All other candidates produced very stable particle sizes across all temperatures tested. The single exception was the SMA/LMA copolymer oligomers, where the waxy nature of the SMA monomer contributed to the high viscosity observed for this oligomer mixture.

This link with the T_g_ values of the materials was supported by the observation that samples exhibiting large particle diameters also tended to show the highest size PDI values under 5 °C storage (Figure 1 bottom), which were lowered by increasing the storage temperature to 25 °C and 40 °C. This was attributed to the majority of materials being well above their T_g_ values at these temperatures and thus likely exhibited reduced viscosity. Furthermore, very good temperature stability (i.e., at ambient and above temperatures) was exhibited by most methacrylate oligomers, which outperformed the shark squalene emulsion. This shows great promise for these materials to be used as adjuvants in emulsion systems that may not need storage at cold temperatures. However, an evaluation of their chemical stability will be necessary to confirm this potential.

### 3.8. In Vitro Innate Immune Stimulation Activity

**Figure 2:**
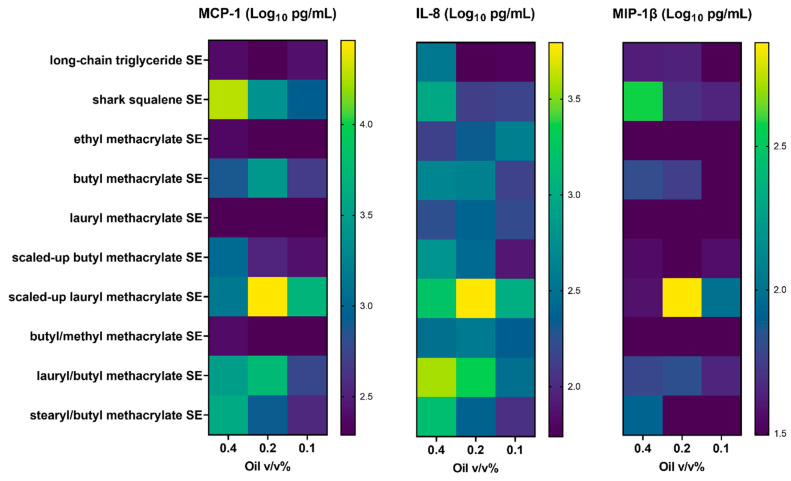
In vitro stimulation of human whole blood with methacrylate-based oil-in-water emulsions resulted in differential cytokine activity. Heat map representation of log-transformed cytokine concentrations measured in supernatants of heparinized blood stimulated by incubation with stable emulsions (SEs). The various oils were evaluated in comparison with shark squalene emulsion (positive control) and long-chain triglyceride emulsion (negative control). Values represent the mean from 6 donors (3 male and 3 female).

The emulsions were then subjected to in vitro assays to determine if they exhibited immunostimulatory properties. Stable emulsions were first evaluated for innate immunostimulatory activity in whole blood from six human subjects by examining cytokine activity (Figure 2).

As expected, the negative control long-chain triglyceride emulsion showed no appreciable immunostimulatory activity, whereas the shark squalene emulsion (positive control) elicited robust cytokine levels in a concentration-dependent manner (Figure 2). Several methacrylate-based emulsions, including the BMA (Table 6, Entry 2), scaled-up BMA (Table 6, Entry 5), scaled-up LMA (Table 6, Entry 6), LMA/BMA (Table 6, Entry 8), and SMA/BMA emulsions (Table 6, Entry 9), resulted in cytokine levels comparable to shark squalene (Figure 2). Interestingly, scaled-up LMA (Table 6, Entry 6) induced high cytokine levels at a 0.2% oil concentration but reduced values at 0.4% oil, which may indicate potential dose-limiting cytolytic activity. This result may indicate that different methacrylate mixtures may exhibit varying parameters, such as solubility, viscosity, aggregation, and/or diffusion, which may influence their behavior as adjuvants.

**Figure 3:**
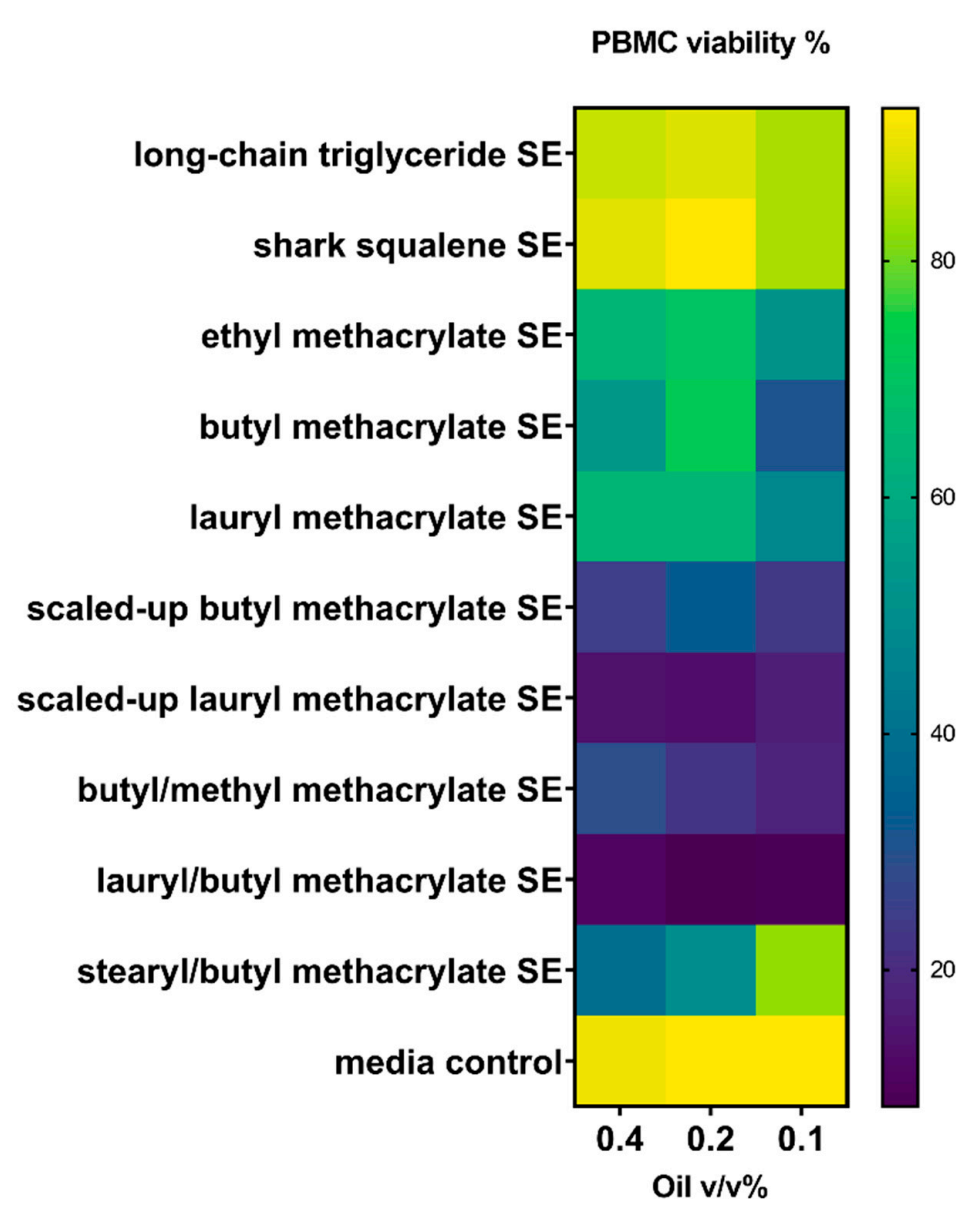
Viability of peripheral blood mononuclear cells (PBMCs) following incubation with methacrylate-based oil-in-water emulsions. PBMCs from a human male donor were employed.

To further assess emulsion biocompatibility, the viability of PBMCs from a human donor was evaluated after incubation with the emulsions (Figure 3).

Indeed, all the methacrylate-mixture-based emulsions decreased PBMC viability compared with the shark squalene or long-chain triglyceride emulsions (Figure 3), with scaled-up LMA (Table 6, Entry 6) and LMA/BMA (Table 6, Entry 8) demonstrating the largest reductions in PBMC viability. BMA (Table 6, Entry 2) and SMA/BMA (Table 6, Entry 9) had a more modest effect on PBMC viability while still eliciting increased cytokine levels (Figure 2 and Figure 3). Since the products of cell necrosis or apoptosis may result in immunostimulatory activity [49,50,51], it is possible that the increased cytokine levels elicited by the methacrylate-mixture-based emulsions are in part due to their cytolytic properties. At the same time, it is apparent that some emulsions that were cytolytic did not induce robust cytokines. Consequently, this is currently the subject of a follow-up study. As these samples were initial polymerization mixtures, these biological responses may in part be due to the presence of free monomers in the oligomer samples. As previously stated, the normal method of purifying small-scale samples was not as effective with these samples due to their physical form and the high boiling point of the monomer. Thus, the next stage of this work will investigate routes to scale up and purify these target materials to determine if purified samples have improved biological performance. Furthermore, the influence of the catalyst residues will also be considered in this scale-up study.

### 3.9. In Vivo Adaptive Immune Stimulation Activity

**Figure 4:**
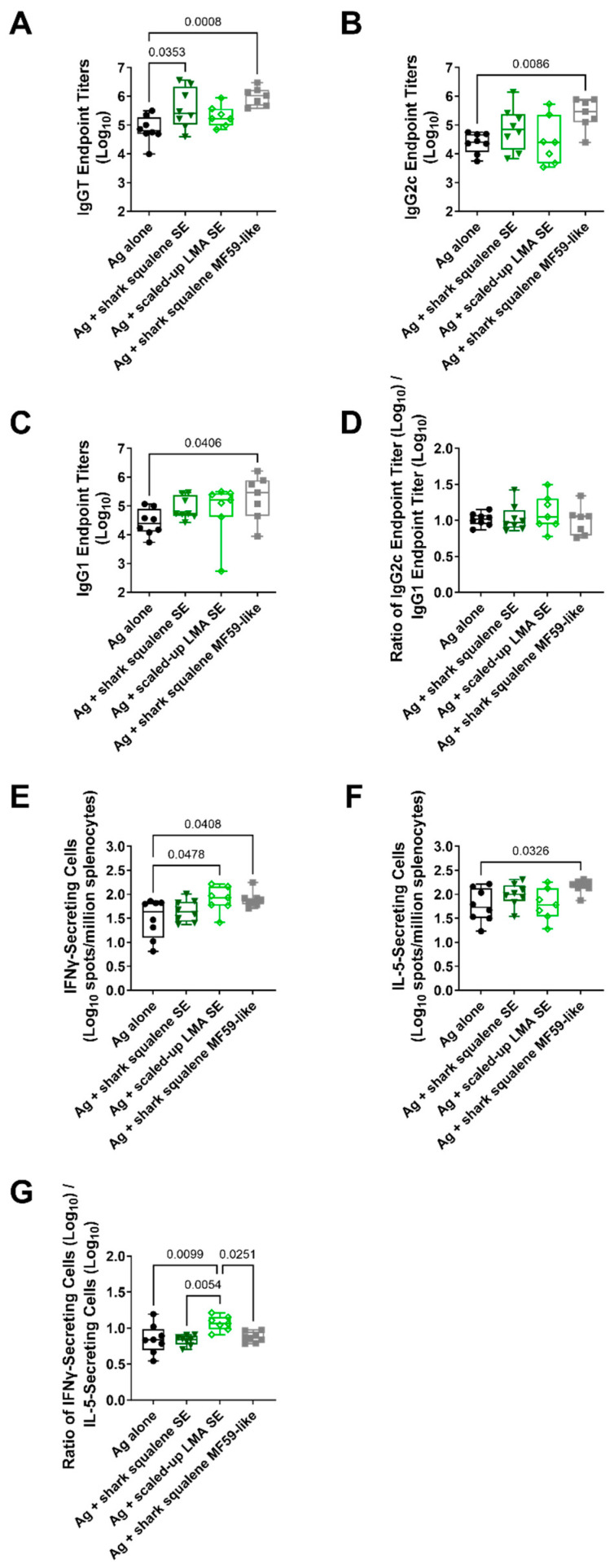
Scaled-up lauryl methacrylate (LMA) emulsion adjuvant activity in mice immunized with split, inactivated H5N1 vaccine antigen. Vaccine antigen (Ag) was mixed with emulsion immediately prior to intramuscular immunization of C57BL/6 (*n* = 7–8) mice. All mice were immunized twice, three weeks apart. Serum and splenocytes were collected three weeks after the 2nd immunization. Data are represented as box-whisker plots, with bars representing median values, boxes representing 1st–3rd quartiles, and whiskers representing the maximum and minimum values. Statistical comparisons were performed for normally distributed data using one-way ANOVA with Tukey’s correction for multiple comparisons, and for non-normally distributed data, using the Kruskal–Wallis test with Dunn’s correction for multiple comparisons, with *p*-values of <0.05 reported on the plots. (**A**) Antigen-specific serum total IgG (IgGT) endpoint titer measured using ELISA. (**B**) Antigen-specific serum IgG2c endpoint titer measured using ELISA. (**C**) Antigen-specific serum IgG1 endpoint titer measured using ELISA. (**D**) Ratio of IgG2c/IgG1 endpoint titers. (**E**) Antigen-specific IFNγ-secreting splenocytes measured using ELISpot. (**F**) Antigen-specific IL-5-secreting splenocytes measured using ELISpot. (**G**) Ratio of IFNγ-secreting cells/IL-5-secreting cells.

To assess the proof of concept for the modulation of adaptive immune responses, a new batch of the emulsion formulation containing the scaled-up LMA (Table 6, Entry 6) was prepared and combined with a split, inactivated pandemic H5N1 influenza vaccine antigen. The emulsion particle size was evaluated before and after mixing to establish that the antigen had no effect on adjuvant physicochemical properties (Figure S11). Mice were immunized twice intramuscularly with the vaccine antigen alone, the vaccine antigen combined with shark squalene emulsion (SE or MF59-like), or the vaccine antigen combined with the scaled-up LMA emulsion. An evaluation of antigen-specific antibody responses indicated that the scaled-up LMA emulsion did not appear to substantially affect serum antibody response magnitudes compared with the vaccine antigen alone, whereas the shark squalene emulsion did induce at least one significantly higher antibody response readout compared with the antigen alone (Figure 4A–D). However, the scaled-up LMA emulsion tended to elicit a higher IFNγ response compared with the antigen alone (*p* = 0.0478), whereas it had no impact on the IL-5 response (Figure 4E,F). In contrast, the shark squalene SE emulsion did not increase either cytokine compared with the antigen alone, and the shark squalene MF59-like emulsion increased both the IFNγ and IL-5 responses compared with the antigen alone. Nevertheless, the IFNγ/IL-5 response ratio was significantly higher in the scaled-up LMA emulsion group compared with the squalene emulsion groups (*p* = 0.0054 and *p* = 0.0251) or the antigen alone group (*p* = 0.0099) (Figure 4G). Thus, it appears that the scaled-up LMA emulsion skews cellular responses to a Th1-type quality, a desirable adjuvant property. However, an assessment of the serum IgG2c/serum IgG1 endpoint titer ratios, which are often interpreted as a surrogate measure of Th1-type immune response, indicated no significant differences between the groups (Figure 4D). Thus, additional studies are needed to optimize dosing as well as elucidate the structure–function relationship of poly(acrylate) emulsion adjuvants.

## 4. Conclusions

This report detailed the first systematic screening of free-radical-produced, methacrylate-based oligomer reaction mixtures as potential alternative vaccine adjuvant components. The oligomer mixtures of all the methacrylate monomer species used were successfully obtained by applying cobalt-based CCTP as a polymerization method, up to a scale of ~20 g. The apparent viscosity of each oligomeric mixture was shown to influence the ability to produce the target emulsion particle size and long-term stability (18 months). This characteristic was successfully used as a tool to predict if the mixture would have the correct formulation properties for vaccine preparation. This mixture property was linked to a combination of the theoretical polymer T_g_, oligomer molecular weight, and conversion achieved. This empirical “tool” successfully predicted that mixtures containing homo-oligomers of BMA or LMA and key co-oligomers including these two monomers would formulate well. Assessing this combination of properties proved more dependable than a direct measurement of viscosity or the oligomer T_g_ with the materials under study due to monomer evaporation or entrapment during analysis. Molecular weight influenced whether it was possible to formulate emulsions, but also the methacrylate oligomers’ larger steric size resulted in emulsions that delivered greater physical stability than shark squalene emulsions at high temperatures. These data suggest that vaccine adjuvant formulations could be produced that could be utilized/stored at higher temperatures. If continuing studies prove this capability, this would be a significant step toward improving the ability to respond to future pandemics. Furthermore, squalene derived from an amaranth plant source was found to generate less physically stable emulsions compared with shark-derived squalene. The in vitro innate immune stimulation activity of the formulated oligomer emulsions showed that poly(LMA) and poly(LMA-BMA) oligomer mixtures demonstrated particularly promising cytokine stimulation activity, and poly(LMA) appeared to elicit Th1-type cellular immune response activity in immunized mice when administered with a vaccine antigen. However, additional studies are merited to define the mechanisms behind these results and optimize the use of these materials as vaccine adjuvants, including identifying which oligomer(s) of the mixtures is responsible for the immunological activity. Thus, this screening study successfully identified key candidates that show potential for application as vaccine adjuvants. By demonstrating that these mixtures can deliver emulsions of an appropriate size, these data support further study into the preparation of oligomer-mixture-based vaccine adjuvants as ethical alternatives to shark-derived squalene. In addition, whilst attempts were made to produce candidate mixtures for screening with iron-based CCTP catalysts, it proved impossible to prepare methacrylate oligomer mixtures of sufficiently low molecular weight to allow for successful vaccine adjuvant formulation. Finally, this study confirmed that microwave heating methods promoted propagation and not chain transfer, as larger molecular weights were obtained compared with conventional heating methods.

## Figures and Tables

**Table 1:** Results from cobalt CCTP and standard free radical control of the oligomerization of methyl methacrylate (MMA) ^1^.

Entry	Solvent	Catalyst	Ligand	Catalyst Loading (ppm)	M_n_ (kg/mol)	Ð	ED_p_	Conv. (%)
1	Toluene	Uncontrolled	None	None	52	1.7	520	86
2	Toluene	PhCoBF	None	600	1.0	1.51	10	56
3	Toluene	PhCoBF	None	6000	0.8	2.57	8	26
4	Toluene	CoBr_2_	DPG	600	0.3	1.5	3	25
5	Toluene	CoBr_2_	DPG	6000	0.4	1.8	4	43
6	Toluene	CoBr_2_	DMG	600	0.3	1.6	3	29
7	Toluene	CoBr_2_	DMG	6000	0.5	1.4	3	38

^1^ The conditions for polymerization were MMA (1:1 *v*:*v* MMA:toluene) with 1 wt% AIBN at 80 °C for 3 h. Abbreviations: CCTP = catalytic chain transfer polymerization, DMG = dimethyl glyoxime, DPG = diphenyl glyoxime, ED_p_ = estimated degree of polymerization, and PhCoBF = bis[(difluoroboryl) diphenylglyoximato]cobalt(II).

**Table 2:** Results from iron-catalyzed CCTP of MMA conducted with a metal-pre-catalyst-to-ligand ratio of 1:1 ^1^.

Entry	Ligand	Catalyst Loading (ppm)	Method	M_n_ (kg/mol)	Ð	EDp	Conv. (%)
1	DIPP	600	Isolated	28	1.7	280	94
2	TMP	600	Isolated	35	1.6	350	86
3	DIPP	600	In situ	27	1.7	270	91
4	TMP	600	In situ	23	1.5	230	91
5	DPG	600	In situ	37	1.6	365	45
6	DMG	600	In situ	31	1.6	307	36
7	DIPP	6000	Isolated	22	1.5	220	74
8	TMP	6000	Isolated	20	1.4	200	62
9	DIPP	6000	In situ	26	1.3	260	53
10	TMP	6000	In situ	27	1.6	270	88
11	DPG	6000	In situ	28.7	1.3	286	33
12	DMG	6000	In situ	27.3	1.8	272	42

^1^ The conditions for polymerization were MMA (1:1 *v*:*v* MMA:toluene) with 1 wt% AIBN and 600 or 6000 ppm of ligand and FeBr_2_ precursor at 80 °C for 3 h. For non-catalyzed free-radical polymerization comparison, see Table 1, Entry 1.

**Table 3:** Results from iron-catalyzed (FeBr_2_ precursor) CCTP of MMA heated via the application of microwave energy, conducted with a metal-pre-catalyst-to-ligand ratio of 1:1 ^1^.

Entry	Catalyst Loading (ppm)	Ligand	Method	M_n_ (kg/mol)	Ð	Conv. (%)
1	600	DIPP	CH	27	1.66	9
2	600	DIPP	MWH	51	1.72	93
3	6000	DIPP	CH	26	1.25	54
4	6000	DIPP	MWH	42	1.60	84
5	600	TMP	CH	23	1.58	91
6	600	TMP	MWH	46	1.73	94
7	6000	TMP	CH	27	1.58	88
8	6000	TMP	MWH	48	1.67	92

^1^ The polymerization conditions were MMA (1:1 *v*:*v* MMA:toluene) with 1 wt% AIBN at 80 °C for 3 h. Abbreviations: CH = conventional heating; MWH = microwave heating. For non-catalyzed free-radical polymerization comparison, see Table 1, Entry 1.

**Table 4:** Optimized conditions for in situ polymerization of methacrylate monomers with FeBr_2_ as the metal precursor and dimethyl (DMG) or diphenyl glyoxime (DPG) as ligands, conducted with a metal-pre-catalyst-to-ligand ratio of 1:1 ^1^.

Entry	Lig.	Catalyst Loading (ppm)	Mono	Solv.	M_n_ (kg/mol)	Ð	ED_p_	Conv. (%)
1	DMG	6000	MMA	Tol	54.6	1.8	547	60
2	DMG	6000	BMA	Tol	42.0	1.7	298	46
3	DMG	6000	LMA	Tol	66.4	1.8	260	25
4	DPG	6000	MMA	Tol	37.1	1.8	370	33
5	DPG	6000	BMA	Tol	27.6	1.7	194	82
6	DPG	6000	LMA	Tol	18.2	1.9	72	88

^1^ The polymerization conditions were methacrylate monomers (1:1 *v*:*v* monomer:toluene) with 1 wt% AIBN at 80 °C, with no pre-stir, for 3 h. For non-catalyzed free-radical polymerization MMA comparison, see Table 1, Entry 1.

**Table 5:** Polymerization of methyl acrylate (MA) with FeBr_2_ and DIPP–diimine ligand in reactions conducted with a metal-pre-catalyst-to-ligand ratio of 1:1 ^1^.

Entry	Catalyst Loading (ppm)	Metal Complex	Ligand	Method	M_n_ (kg/mol)	Ð	Conv. (%)
1	0	None	--	Uncontrolled	--	--	Trommsdorf ^2^
2	600	PhCoBF	--	Isolated	--	--	Trommsdorf ^2^
3	6000	PhCoBF	--	Isolated	--	--	Trommsdorf ^2^
4	600	FeBr_2_	DIPP	Isolated	17	2.37	91
5	6000	FeBr_2_	DIPP	Isolated	26	1.61	30
6	600	FeBr_2_	DIPP	In situ	17	3.0	74

^1^ The polymerization conditions were MA (1:1 *v*:*v* MA:toluene) with 1 wt% AIBN at 80 °C for 3 h. The reactions were compared with reactions conducted with the standard catalyst PhCoBF. ^2^ Trommsdorff–Norrish effect refers to an auto-acceleration of the polymerization that occurs due to the onset of diffusion-controlled limitations for the growing radicals to terminate, resulting in a significant decrease in the termination rate, the generation of longer polymer molecules, and a faster polymerization rate. Practically, its onset was noted by a large exotherm and the formation of a “gel”, leading to loss of agitation in the reactor [43,44,45,46,47].

**Table 6:** Summary of oligomer mixtures advanced to evaluation for vaccine adjuvant application and their synthesis via bulk polymerization conducted with a metal-pre-catalyst-to-ligand ratio of 1:1.

E	Cat.	Lig.	Cat. Load (ppm)	AIBN (ppm)	Mono	Scale (mL)	M_n_ (kg/mol)	Ð	ED_p_	Conv. (%)
1	PhCoBF	--	120	10,000	EMA	11	0.55	1.3	5	51
2	PhCoBF	--	120	10,000	BMA	11	0.72	1.3	5.0	42
3	PhCoBF	--	120	10,000	LMA	11	0.97	1.7	4.0	60
4	CoBr_2_	DPG	250	1930	MMA	11	2.4	2.4	23.0	54
5	CoBr_2_	DPG	2500	10,000	Scaled BMA	22	0.5	1.3	3.5	56
6	CoBr_2_	DPG	6000	10,000	Scaled LMA	21	1.72	1.4	7.0	55
7	CoBr_2_	DPG	2500	10,000	BMA/ MMA	33	0.3	1.9	1.5	62
8	CoBr_2_	DPG	2500	7000	LMA/ BMA	34	0.6	2.2	1.5	35
9	CoBr_2_	DPG	2500	7000	SMA/ BMA	35	1.9	2.6	4.0	76
10	CoBr_2_	DPG	2500	7000	SMA/ LMA	23	0.7	1.4	1.5	55

## Data Availability

The data presented in this study are available on request from the corresponding author.

## Supplementary Materials

The following supporting information can be downloaded at https://www.mdpi.com/article/10.3390/polym15183831/s1: Figure S1. Molecular structures of the monomers used in this study: methyl methacrylate (MMA), butyl methacrylate (BMA), ethyl methacrylate (EMA), lauryl methacrylate (LMA), and stearyl methacrylate (SMA); Figure S2. Representative GPC trace for oligomers produced via the cobalt-catalyzed reactions of MMA polymerization; Figure S3. Proposed structures of the catalysts, including the ligands L1 = *N*,*N*-bis(2,6- diisopropylphenyl)ethane-1,2-diimine (DIPP), L2 = *N*,*N*-bis(2,4,6-trimethylphenyl)ethane-1,2-diimine (TMP), L3 = dimethyl glyoxime (DMG), or L4 = diphenyl glyoxime (DPG), used in iron-catalyzed CCTP; Figure S4. Representative GPC trace for oligomers produced via the cobalt-catalyzed reactions of BMA polymerization; Figure S5. Representative GPC trace for oligomers produced via the cobalt-catalyzed reactions of LMA polymerization; Figure S6. Representative GPC trace for oligomers produced via the cobalt-catalyzed reactions of BMA/MMA (50/50) copolymerization; Figure S7. Representative GPC trace for oligomers produced via the cobalt-catalyzed reactions of LMA/BMA (50/50) copolymerization; Figure S8. Representative GPC trace for oligomers produced via the cobalt-catalyzed reactions of BMA/SMA (50/50) copolymerization; Figure S9. Representative GPC trace for oligomers produced via the cobalt-catalyzed reactions of SMA/LMA (50/50) copolymerization; Figure S10. Representative dynamic light scattering intensity-based size distributions of emulsions; Figure S11. Physicochemical stability of emulsion after mixing with H5N1 vaccine antigen and stored at 5 °C or 25 °C for 4 h.
