# Supplementary material for: Screening of Oligomeric (Meth)acrylate Vaccine Adjuvants Synthesized via Catalytic Chain Transfer Polymerization
Source: Polymers (Basel). 2023 Sep 20;15(18):3831. doi: 10.3390/polym15183831 (PMC10538096; doi:10.3390/polym15183831)
Supplement: Supplementary file 1 [file polymers-15-03831-s001.zip › polymers-2564915-supplementary.pdf]

SUPPLEMENTARY MATERIALS

Supplementary Figures

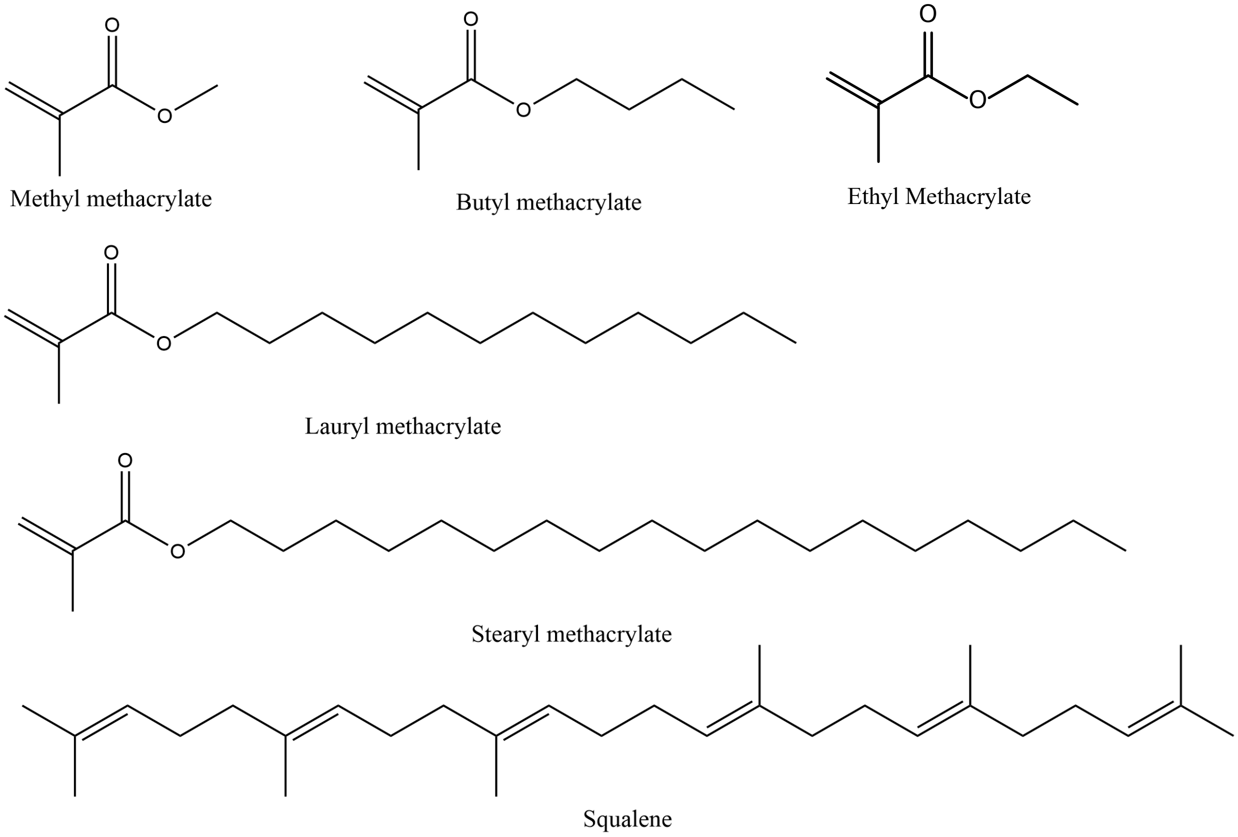

Figure S1. Molecular structures of the monomers used in this study: methyl methacrylate (MMA), butyl methacrylate (BMA), ethyl methacrylate (EMA), lauryl methacrylate (LMA), and stearyl methacrylate (SMA). The figure also shows the molecular structure of squalene.

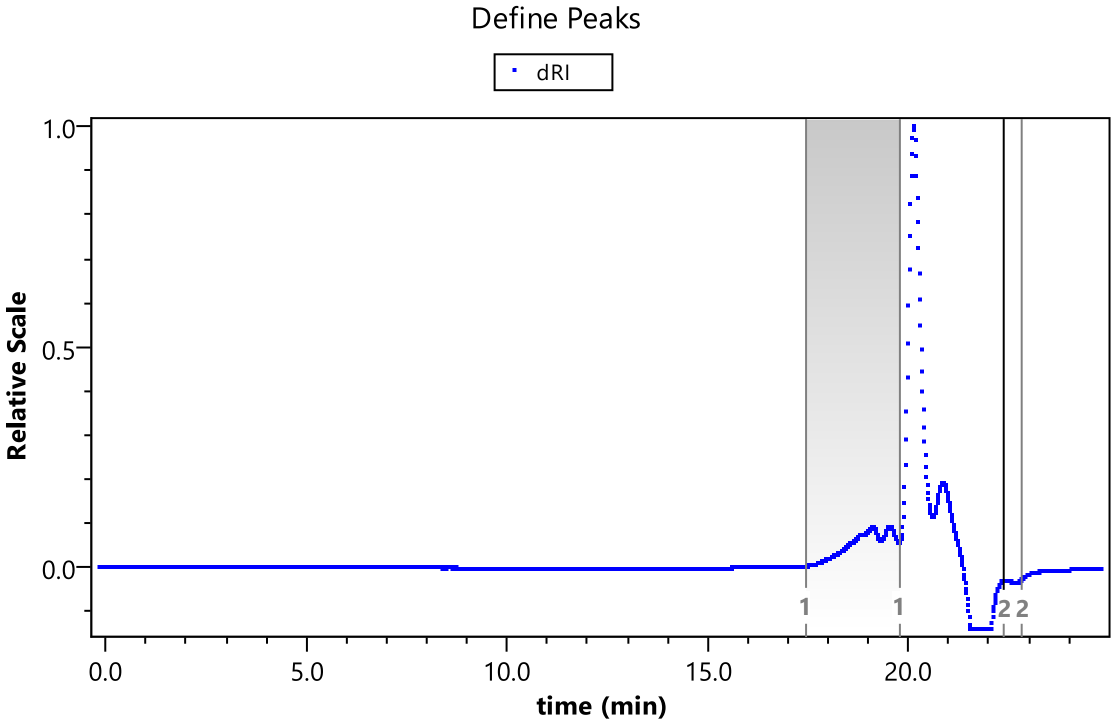

Figure S2. Representative GPC trace for oligomers produced by the cobalt-catalyzed reactions of MMA polymerization. The polymer was generated with $CoBr_2$ as catalyst using DPG ligand and catalyst loading at 600 PPM with 1 wt% AIBN (see Table 1, Entry 4): M_w = 0.43 kg mol^{-1}, M_n = 0.34 kg mol^{-1}, and Đ = 1.3.

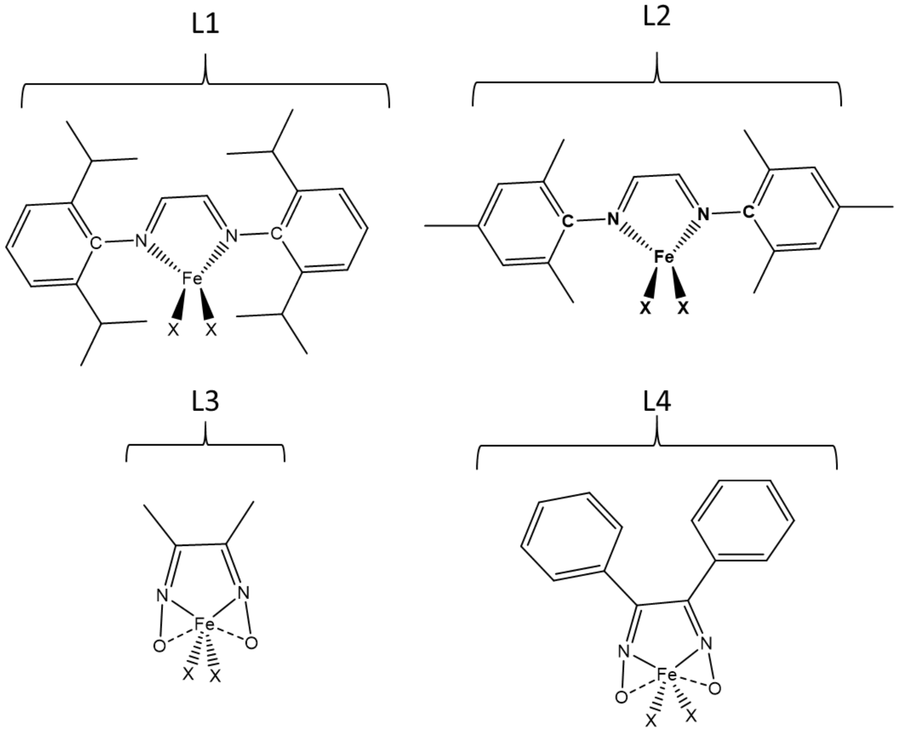

Figure S3. Proposed structures of the catalysts, including the ligands L1 = *N,N*-bis(2,6-diisopropylphenyl)ethane-1,2-diimine (DIPP), L2 = *N,N*-bis(2,4,6-trimethylphenyl)ethane-1,2-diimine (TMP), L3 = dimethyl glyoxime (DMG), or L4 = diphenyl glyoxime (DPG), used in iron-catalyzed CCTP.

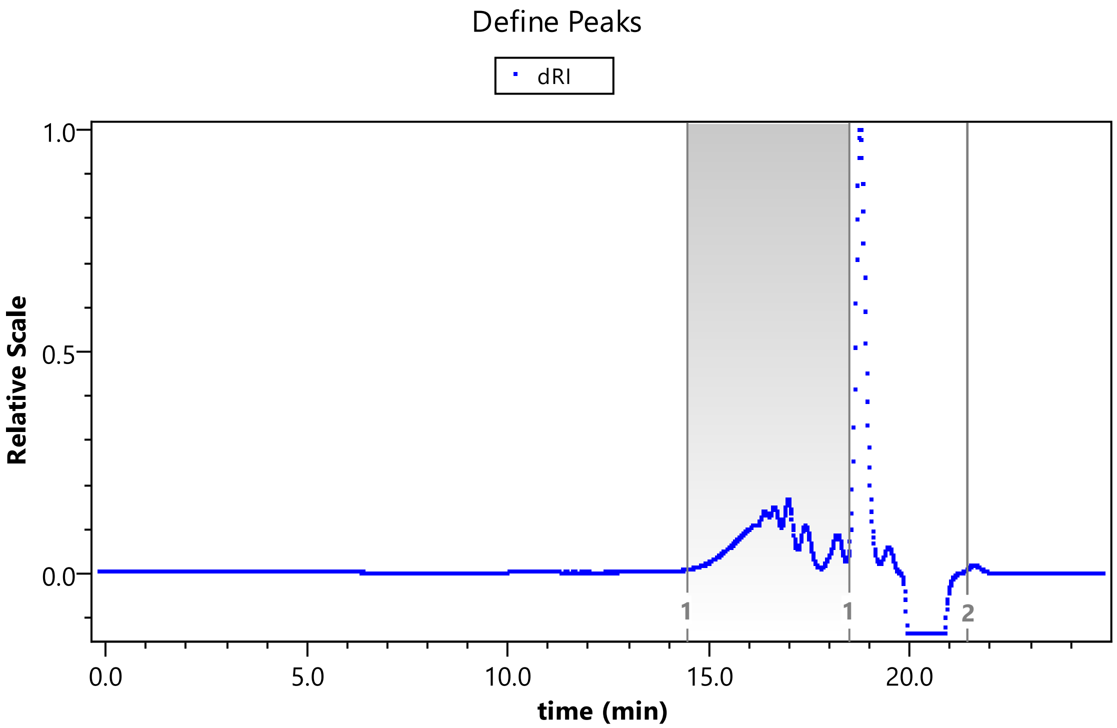

Figure S4. Representative GPC trace for oligomers produced by the cobalt-catalyzed reactions of BMA polymerization. The polymer was generated with $CoBr_2$ as catalyst using DPG ligand and catalyst loading at 2500 PPM with 1 wt% AIBN (see Table 6, Entry 5): M_w = 0.62 kg mol^{-1}, M_n = 0.48 kg mol^{-1}, and Đ = 1.3.

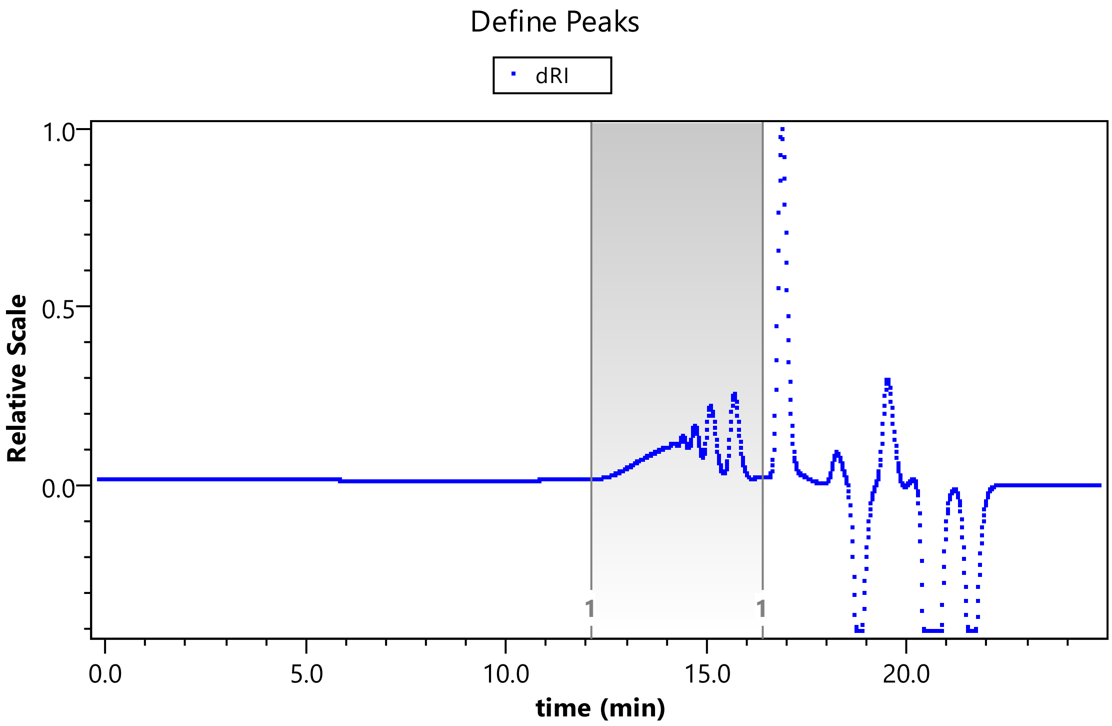

Figure S5. Representative GPC trace for oligomers produced by the cobalt-catalyzed reactions of LMA polymerization. The polymer was generated with $CoBr_2$ as catalyst using DPG ligand and catalyst loading at 6000 PPM with 1 wt% AIBN (see Table 6, Entry 6): M_w = 2.48 kg mol^{-1}, M_n = 1.71 kg mol^{-1}, and Đ = 1.4.

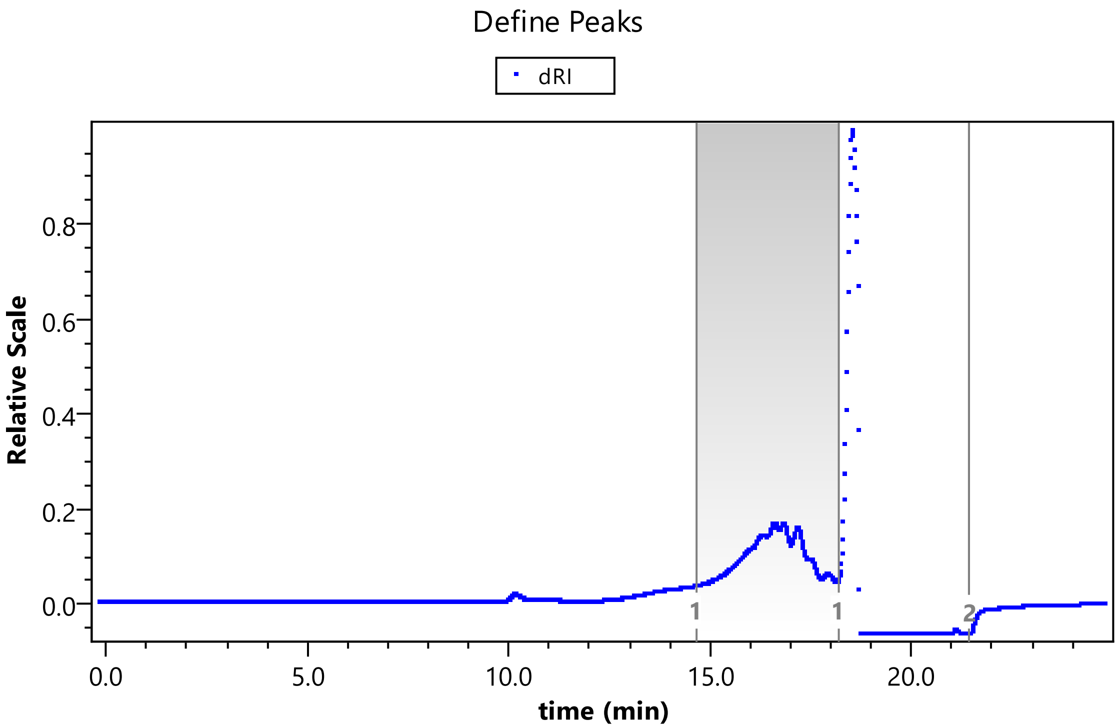

Figure S6. Representative GPC trace for oligomers produced by the cobalt-catalyzed reactions of BMA/MMA (50/50) copolymerization. The polymer was generated with $CoBr_2$ as catalyst using DPG ligand and catalyst loading at 2500 PPM with 1 wt% AIBN (see Table 6, Entry 7): M_w = 0.50 kg mol^{-1}, M_n = 0.30 kg mol^{-1}, and Đ = 1.9.

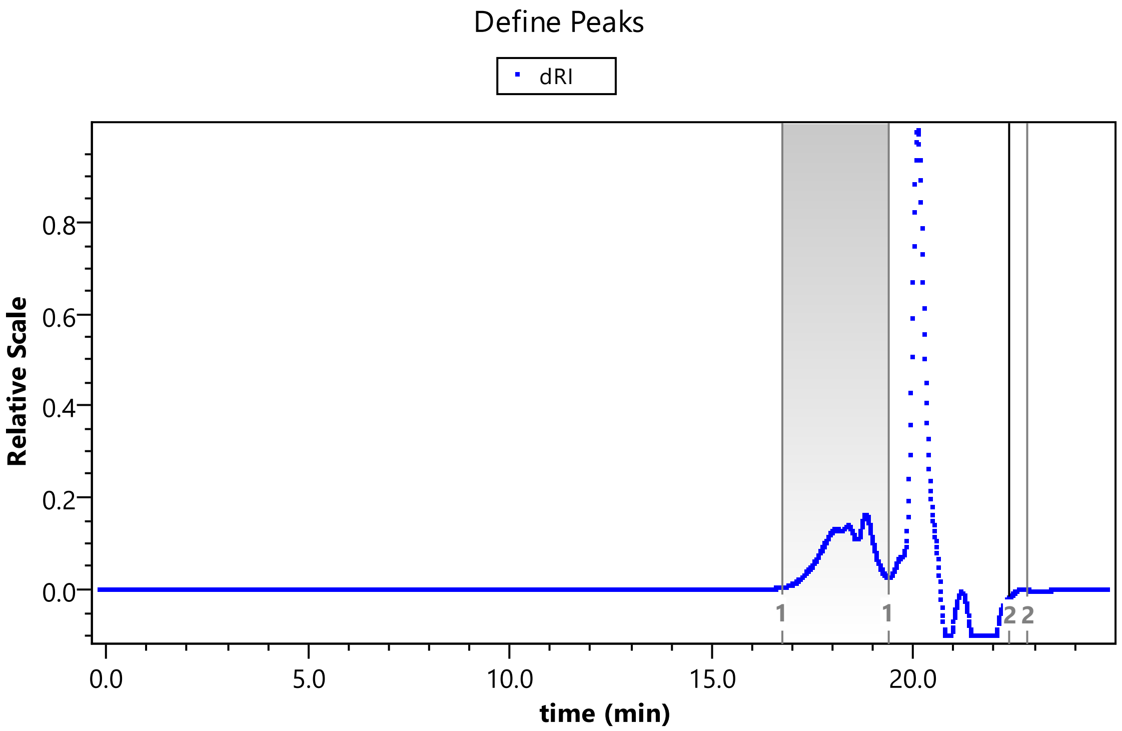

Figure S7. Representative GPC trace for oligomers produced by the cobalt-catalyzed reactions of LMA/BMA (50/50) copolymerization. The polymer was generated with $CoBr_2$ as catalyst using DPG ligand and catalyst loading at 2500 PPM with 0.7 wt% AIBN (see Table 6, Entry 8): M_w = 1.35 kg mol^{-1}, M_n = 0.6 kg mol^{-1}, and Đ = 2.2.

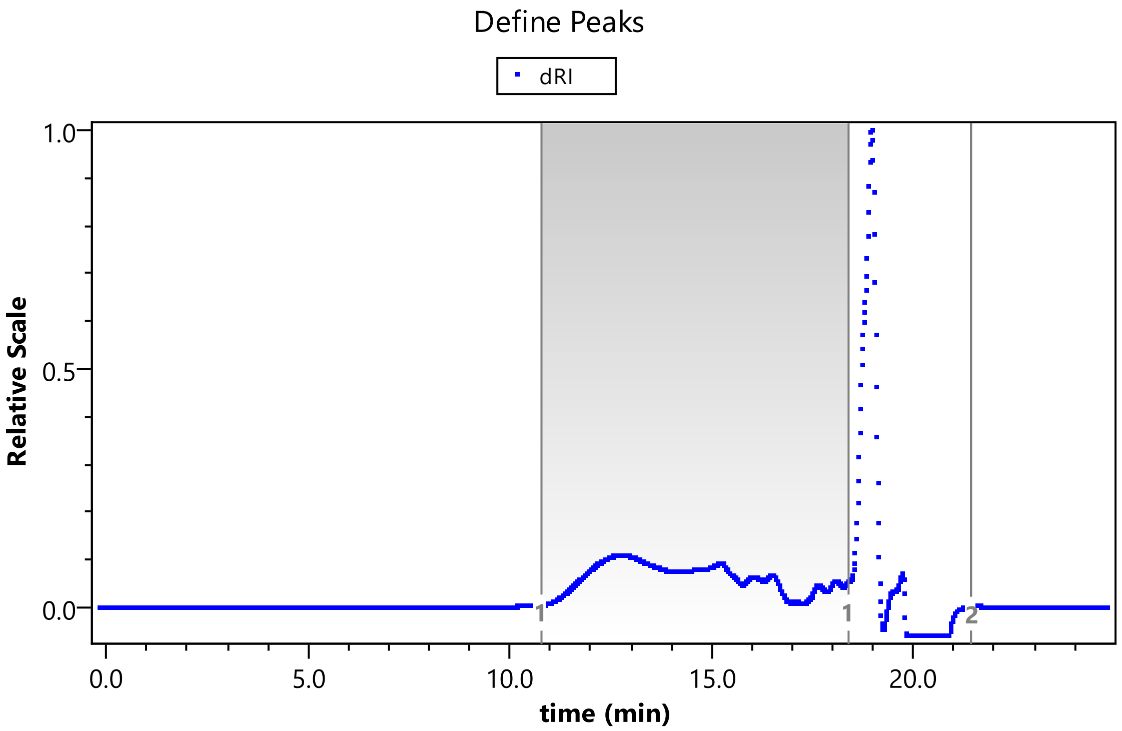

Figure S8. Representative GPC trace for oligomers produced by the cobalt-catalyzed reactions of BMA/SMA (50/50) copolymerization. The polymer was generated with $CoBr_2$ as catalyst using DPG ligand and catalyst loading at 2500 PPM with 0.7 wt% AIBN (see Table 6, Entry 9): $M_w = 4.91$ kg mol^{-1}, $M_n = 1.9$ kg mol^{-1}, and Đ = 2.6.

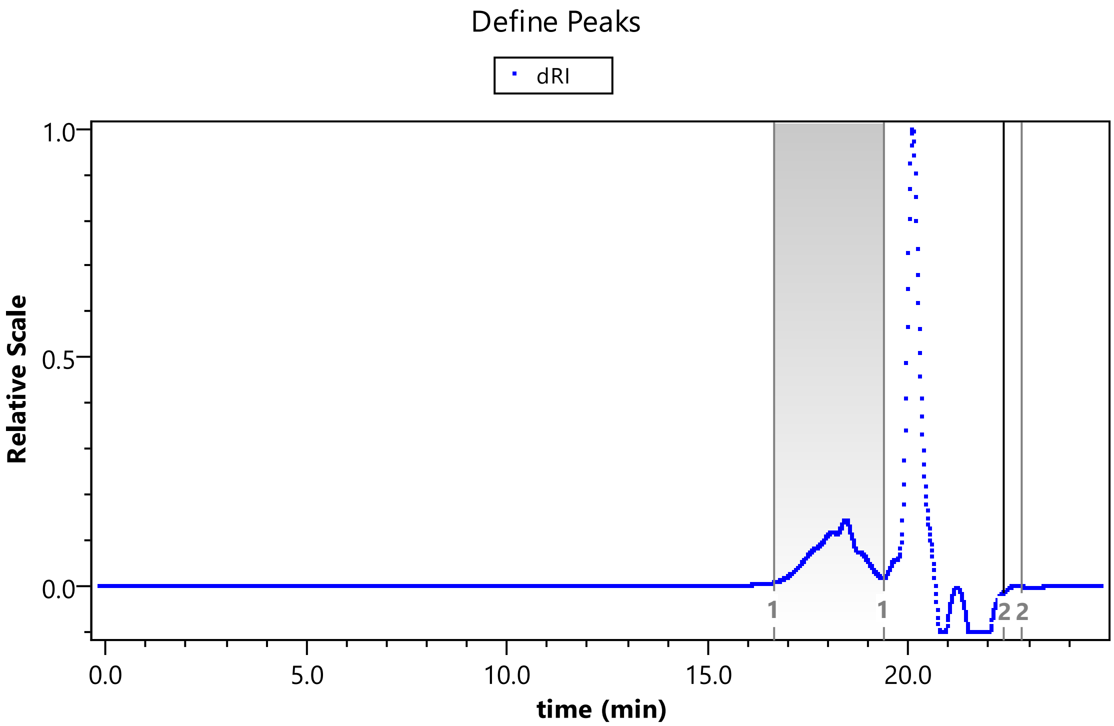

Figure S9. Representative GPC trace for oligomers produced by the cobalt-catalyzed reactions of SMA/LMA (50/50) copolymerization. The polymer was generated with $CoBr_2$ as catalyst using DPG ligand and catalyst loading at 2500 PPM with 0.7 wt% AIBN (see Table 6, Entry 10): $M_w = 0.97$ kg mol^{-1}, $M_n = 0.70$ kg mol^{-1}, and $Đ = 1.4$.

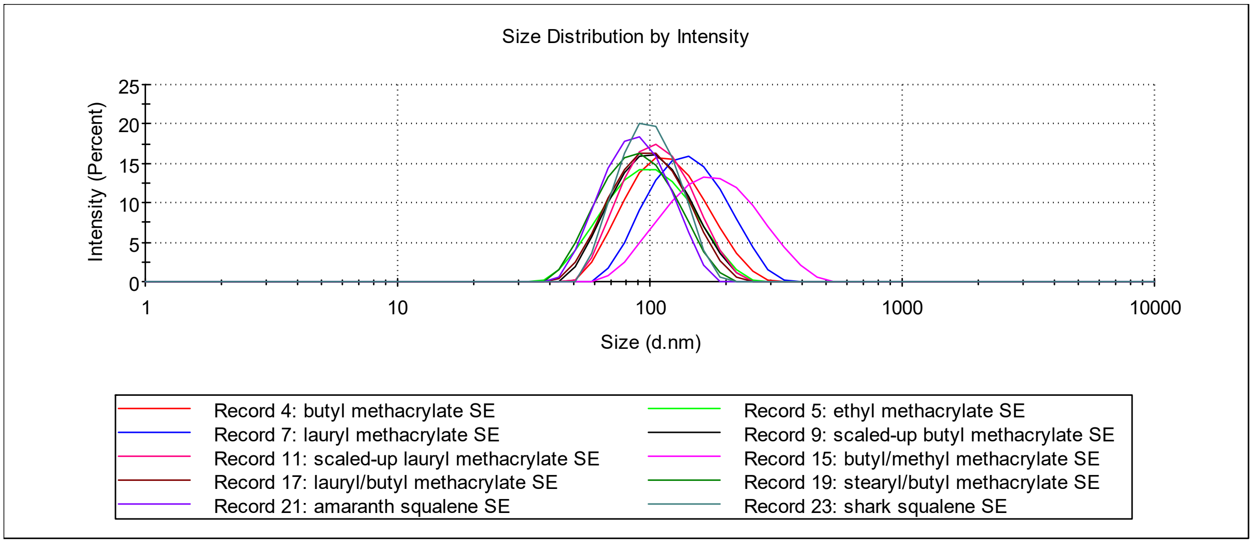

Figure S10. Representative dynamic light scattering intensity-based size distributions of emulsions. Measurements were conducted immediately following emulsion manufacture.

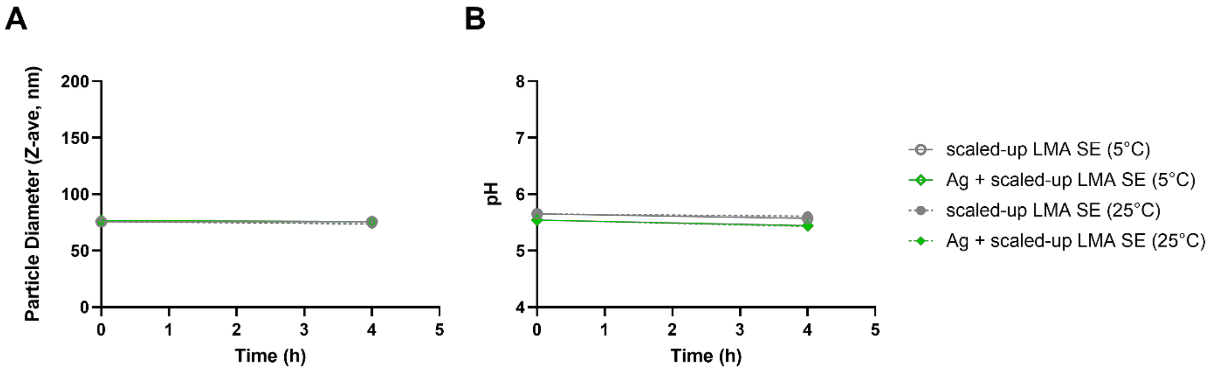

Figure S11. Physicochemical stability of emulsion after mixing with H5N1 vaccine antigen and stored at 5°C or 25°C for 4 hours. A) Formulation particle diameter measured by dynamic light scattering. B) Formulation pH. Ag = antigen, LMA = lauryl methacrylate, SE = stable emulsion.
